# Supplementary material for: Content-rich biological network constructed by mining PubMed abstracts
Source: BMC Bioinformatics. 2004 Oct 8;5:147. doi: 10.1186/1471-2105-5-147 (PMC528731; doi:10.1186/1471-2105-5-147)
Supplement: Additional File 2 — The original results of the above study (non-essential files are deleted to keep the file size under the limit set by BMC bioinformatics). [file 1471-2105-5-147-S2.bz2 › chilibotAdditionalFile2/dip20/706ID10734142E18043/html/MUTS_UVRD.html]

 


 **MUTS** and **UVRD** 
  
Found 31 abstracts in PubMed, retrieved 20.  
 

 What does Google say? 
 PDF only 
| .edu only 

---

**Interactive relationship** (e.g. stimulation, inhibition, etc)

**Inhibitory relationship**- This phenotype is suppressed by a  **uvrD**  mutU defect, and partially suppressed by mutH, mutL, or  **mutS**  mutations.  Ref: 11381137 Proc Natl Acad Sci U S A, 2001
**Neutral relationship**- **MutS**  and MutL activate DNA helicase II  [ **UVRD** ]  in a mismatch dependent manner.  Ref: 9535910 J Biol Chem, 1998
- All possible pairwise combinations of  **UvrD** , MutL,  **MutS** , and MutH were tested using the yeast two hybrid system to identify potential interactions involving mismatch repair proteins.  Ref: 9880500 J Biol Chem, 1999

**Non-interactive relationship** (e.g. studied together, co-existance, homology, etc.)

- Escherichia coli  **MutS** , MutL, and DNA helicase II  [ **UVRD** ]  are sufficient to initiate mismatch dependent unwinding of an incised heteroduplex Yamaguchi, M.  Ref: 9535911 J Biol Chem, 1998
- The increases in spontaneous reversion of histidine mutations were first measured in isogenic strains carrying mismatch repair defective mutH, mutL,  **mutS** , or  **uvrD**  alleles, relative to their mismatch repair proficient counterparts.  Ref: 9685594 Mutat Res, 1998
- Deletion rates were enhanced 30 to 50 fold by the mismatch repair defects,  **mutS** , mutL and  **uvrD** , and 250 fold by the ssb 3 allele, but the effect of the mismatch defects was canceled by the delta recA allele.  Ref: 9055067 Genetics, 1997
- We screened the following host mutator mutations for effects on spontaneous mutation rates in T4 mutT destruction of aberrant dGTPs, polA, polB and polC DNA polymerases, dnaQ exonucleolytic proofreading, mutH,  **mutS** , mutL and  **uvrD**  methyl directed DNA mismatch repair, mutM and mutY excision repair of oxygen damaged DNA, mutA function unknown, and topB and osmZ affecting DNA topology.  Ref: 7851754 Genetics, 1994
- We also describe the alterations in the  **mutS** , mutL and  **uvrD**  genes responsible for the mutator phenotype of hypermutable P. aeruginosa strains isolated from CF patients.  Ref: 11952911 Mol Microbiol, 2002
- We present novel phylogenetic evidence for horizontal transfer of three genes involved in DNA replication and repair  **mutS** ,  **uvrD** , and polA.  Ref: 11746762 Environ Mol Mutagen, 2001
- **MutS** , MutL, and DNA helicase II  [ **UVRD** ]  are required for the mismatch provoked excision step that occurs during Escherichia coli methyl directed mismatch repair.  Ref: 9535910 J Biol Chem, 1998
- As judged by protein affinity chromatography, MutL and MutL E32K both support formation of ternary complexes that also contain  **MutS**  and MutH or  **MutS**  and DNA helicase II  [ **UVRD** ] .  Ref: 10734142 J Biol Chem, 2000
- The in vitro activity was also dependent on products of mutH, mutL,  **mutS** , and  **uvrD**  loci and was equally efficient on nucleotide insertions and deletions.  Ref: 9278429 J Biol Chem, 1997
- **MutS**  and MutL dependent activation of DNA helicase II  [ **UVRD** ]  has also been demonstrated with a model substrate in which a 138 residue oligonucleotide was hybridized to a 138 nucleotide gap in an otherwise duplex 00 base pair circular DNA.  Ref: 9535910 J Biol Chem, 1998
- By genetic analysis, we show that the inhibitory effect of ssDNA exonucleases on deletion formation is independent of the  **MutS**  and  **UvrD**  proteins.  Ref: 12538867 Proc Natl Acad Sci U S A, 2003
- Seven out of the 11 mutator strains were found to be defective in the MMR system four  **mutS** , two mutL and one  **uvrD** .  Ref: 11952911 Mol Microbiol, 2002
- Repair of both heteroduplex orientations requires MutH, MutL,  **MutS** , DNA helicase II  [ **UVRD** ] , SSB, and DNA polymerase III holoenzyme, but the two substrates differ with respect to exonuclease requirements for correction.  Ref: 8389365 J Biol Chem, 1993
- The results shown here indicate that the putative P. aeruginosa  **mutS** , mutL and  **uvrD**  genes are mutator genes.  Ref: 11952911 Mol Microbiol, 2002
- Unwinding of both substrates in the presence of  **MutS** , MutL, DNA helicase II  [ **UVRD** ] , and single stranded DNA binding protein was mismatch dependent and initiated at the single strand break.  Ref: 9535911 J Biol Chem, 1998
- Displacement of the oligonucleotide requires  **MutS** , MutL, DNA helicase II  [ **UVRD** ] , and ATP and is dependent on the presence of a mismatch within the hybrid region.  Ref: 9535910 J Biol Chem, 1998
- The mismatch repair system  **mutS** , mutL and  **uvrD**  genes in Pseudomonas aeruginosa molecular characterization of naturally occurring mutants.  Ref: 11952911 Mol Microbiol, 2002
- In Test A, repair proficient strain and its uvrA counterpart are used for detection of spontaneous and UV induced mutations, while in Test B mismatch repair deficient strains mutH,  **mutS** , mutL and  **uvrD**  are used for amplified detection of spontaneous mutations caused by replication errors.  Ref: 9675243 Mutat Res, 1998
- The growth defect and filamentation phenotypes associated with 2 aminopurine exposure are effectively suppressed by null mutations in mutH, mutL,  **mutS** , or  **uvrD**  mutU, which encode activities that act upstream of the four exonucleases in the mechanism for the methyl directed reaction that has been proposed based on in vitro studies.  Ref: 11381137 Proc Natl Acad Sci U S A, 2001
- To investigate the effects on PV rates of mutations in genes for mismatch repair MMR, insertion deletion mutations of  **mutS** , mutL, mutH, dam, polI,  **uvrD** , mfd and recA were constructed in Hi strain Rd.  Ref: 11889052 EMBO J, 2002
- The Escherichia coli methyl directed reaction has been reconstituted in a purified system consisting of MutH, MutL, and  **MutS**  proteins, DNA helicase II  [ **UVRD** ] , single strand DNA binding protein, DNA polymerase III holoenzyme, exonuclease I, DNA ligase, along with ATP adenosine triphosphate, and the four deoxynucleoside triphosphates.  Ref: 2665076 Science, 1989
